# Desmoid tumors complicating Familial Adenomatous Polyposis: a meta-analysis mutation spectrum of affected individuals

**DOI:** 10.1186/s12876-015-0306-2

**Published:** 2015-07-16

**Authors:** Voytek Slowik, Thomas Attard, Hongying Dai, Raj Shah, Seth Septer

**Affiliations:** 1 Section of Pediatric Gastroenterology, Children’s Mercy Hospital, Kansas City, MO USA; 2 Department of Medical Research, Children’s Mercy Hospital, Kansas City, MO USA; 3 University of Missouri Kansas City – School of Medicine, Kansas City, MO USA

**Keywords:** Familial adenomatous polyposis, FAP, Desmoid tumors, Solid tumors, Intra-abdominal tumors, Adenomatous polyposis gene mutation, APC

## Abstract

**Background:**

Desmoid tumors are a group of benign, invasive, solid tumors that are relatively rare in the general population, but can occur in up to 21 % of patients with Familial Adenomatous Polyposis (FAP). They can be difficult to treat and have high rates of recurrence even after resection. Our goal with this study was to identify the genetic mutations that put certain patients with FAP at high risk for desmoid tumors and could be future targets for research.

**Methods:**

We performed a search in Pubmed, Ovid Medline and Embase to identify subjects with desmoid tumors and FAP. As a reference group for APC mutations in the unselected FAP population, we used the UMD-APC database referenced in the Orphanet portal which includes APC mutation data on 2040 individuals with FAP.

**Results:**

Mutations were able to be broken down into 7 regions based on previously published data. Mutations in the APC gene from codons 1310 to 2011 were the most common region encompassing 48 % of published desmoid cases and 40 % of the reference population. It had a slightly elevated odds ratio of 1.4 that was statistically significant along with codon region 543-713 that had an odds ratio of 2.0. Using a combination of p-value and CI, the remaining 5 regions did not meet statistical significance as either the p >0.05 or the CI included 1.0. The most common point mutation found was codon 1309 (13.1 %), but it was also the most commonly found mutation in our reference population (12.9 %) and had an odds ratio of 1.0.

**Conclusions:**

There is an increased risk for desmoid tumors in individuals with APC mutations between codons 543-713 and 1310-2011 when compared to a reference population. These patients may benefit from further study to develop surveillance protocols that could improve outcomes.

**Electronic supplementary material:**

The online version of this article (doi:10.1186/s12876-015-0306-2) contains supplementary material, which is available to authorized users.

## Background

Familial Adenomatous Polyposis (FAP) is an autosomal dominant syndrome characterized by profuse adenomatous polyposis in the colon and rectum with nearly 100 % life- time risk of colorectal cancer [1]. Germline mutations with frameshift or nonsense codes in the adenomatous polyposis coli (APC) gene on chromosome 5q21 cause the majority of cases of FAP [2]. More than 60 % of APC mutations are found in the mutation cluster region(MCR) between codons 1284 and 1580 [3], or 1284–1464 [4]. The two most frequently described germline mutations are located at codon 1309 (c3927_3931delAAAGA) and codon 1061 (c.3183_87delACAAA) [5].

Penetrance is variable for extra-colonic manifestations, but contributes significantly to the morbidity and mortality of patients post-colectomy. Disease expression in FAP is to some extent dependent on the specific APC mutations and all patients identified with FAP need to be assessed regularly from birth. Extra-colonic manifestations in children with FAP may include an increased risk of hepatoblastoma [6, 7], medulloblastoma [8], osteomas, supernumerary teeth or missing teeth, congenital hypertrophy of retinal pigment epithelium (CHRPE), desmoid tumors, thyroid cancers, and fibromas. Several referral centers have adopted an extra-intestinal tumor surveillance strategy that includes reported genotype-phenotype correlations [9, 10].

Desmoid tumors are a significant concern in patients post-colectomy and cause significant morbidity and mortality [11]. The overall lifetime risk in some patient registries of FAP for desmoids tumors is as high as 21 % [12, 13]. Risk factors include prior surgery, positive family history, osteomas, and epidermoid cysts [12]. Despite their common occurrence in FAP, they are poorly understood, histologically benign lesions. They are not associated with distant metastases, but can cause significant morbidity from local tissue compression from growth. Symptoms generally depend on the location of the tumor and can range from asymptomatic to aesthetic changes, pain, bowel obstruction, musculoskeletal dysfunction, abscess formation, and vascular compression.

Previously, it was felt that the risk for desmoids tumors was most severe in patients with FAP whose mutations were between codons 1444 and 1578 [12]. However, this was based on observations on detected mutations in affected patients without comparison to a reference population.

This study is a systematic review of the genotype-phenotype associations of desmoids tumors in patients with FAP (Additional file 1). A literature search was undertaken to find all cases of desmoids tumors that had a described mutation in the APC gene. This was further refined by controlling for the frequency distribution of mutations in a reference database of APC gene mutations in FAP patients. The aim of this study is to better define the correlation between APC gene mutations and desmoid tumor formation. This could provide future targets of research for pathophysiology and surveillance programs.

## Methods

All authors performed a search in Pubmed, Ovid Medline and Embase with the two terms:

- ((“Adenomatous Polyposis Coli”[Mesh] OR “Genes, APC”[Mesh] OR “Adenomatous Polyposis Coli Protein”[Mesh]) AND “Genetic Phenomena”[mesh]) AND “Epidemiologic Studies”[Mesh] AND (“Fibroma”[Mesh] OR desmoid) AND English[lang].
- (“Fibroma”[Mesh] OR desmoid) AND ((kindred* OR family OR “Family”[Mesh] OR “Pedigree”[Mesh]) AND (“Adenomatous Polyposis Coli”[Mesh] OR “Genes, APC”[Mesh] OR “Adenomatous Polyposis Coli Protein”[Mesh]) AND “Genetic Phenomena”[mesh] AND English[lang]

Our search results were reviewed by all authors. Articles in the reference lists of the retrieved articles were also reviewed to identify additional studies. All of the authors collectively abstracted information from each of these studies. Each published case was reviewed for data points including sample size, diagnosis of FAP, diagnosis of desmoids tumor, and documentation of APC mutation. Studies with duplicate patient reports were excluded. The remaining patients were accrued into a database where information relating to gender, age of onset for desmoid tumors, desmoid tumor location, and specific mutations of APC gene were collected for further evaluation. As a reference group for APC mutations in the unselected FAP population, we used the UMD-APC database referenced in the Orphanet portal (http://www.umd.be/APC/). This includes data on 2040 individuals with FAP including their APC gene mutation.

When reviewing the published literature on desmoid tumors, we found that many studies did not report specific point mutations of the APC gene. Instead, several studies grouped patients together into ranges of codon mutations. These ranges were often based on the results of protein truncation tests commonly used to identify mutations in the APC gene prior to sequencing. The largest of the ranges was from a study by Bertario et al and this was used as the basis for our statistical analysis [4]. Given the rarity of both desmoid tumors and FAP, the decision was made to include these patients into the database in order to have as many published cases as possible. While this would limit our ability to analyze specific codon mutations, we are able to increase the statistical significance for the codon mutation ranges. One codon was able to be fully analyzed as a point mutation, 1309, and the results of that analysis are included below.

Fisher’s exact test was performed to compare the mutation rate between reference population and patients with FAP and desmoids tumors. Odds ratio and 95 % exact confidence interval were determined. The 95 % confidence interval of significant odds ratio does not cross 1. Statistical significance was claimed with *p* < 0.05. All statistical analyses were performed in SAS 9.2 (Cary NC).

## Results

**Fig. 1:**
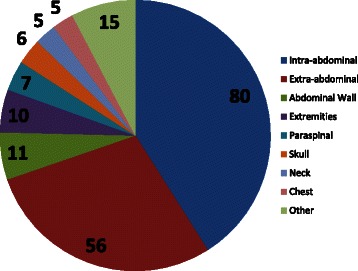
Pie chart showing published locations of desmoids tumors associated with FAP (n = 195). The exact location of extra-abdominal tumors are included in the figure when reported, but they were often simply reported as “extra-abdominal”

The initial searches resulted in 51 unique articles related to desmoids and FAP. After review, twenty six studies were determined as meeting inclusion criteria. These twenty six studies included a total of 274 reported cases of desmoids tumors in patients with FAP. APC gene mutation testing was reported in 222 patients [Table 1]. Of those 222 cases, individuals were mainly female (63 F: 43 M) with an average age of 30.3 (+/− 27.0) years. The reference population of individuals with FAP included 2040 individuals. We compared the prevalence of APC mutations in the published patients with desmoid tumors to the prevalence of the same mutation in the reference population [Table 1]. The single most common location for a desmoid tumor was intra-abdominal; however, in total there were more extra-abdominal tumors with the next two most common locations being the abdominal wall itself and the extremities [Fig. 1].

**Table 1:** Codon regions and their respective odds ratios for risk of desmoids tumors

Codon	Number in Published Articles (222)	% in Published Population	Number in Reference Database (2043 total)	% in Reference Group	Odds Ratio (CI)	P-value
159-495	23	10 %	197	10 %	1.1 (0.7-1.7)	0.72
543-713	21	9 %	102	5 %	2.0 (1.2-3.3)	0.01
721-972	19	9 %	105	5 %	1.7 (1.0-2.9)	0.04
976-1067	19	9 %	184	9 %	0.9 (0.5-1.6)	0.9
1068-1237	19	9 %	150	7 %	1.2 (0.7-2.0)	0.5
1256-1303	14	6 %	65	3 %	2.0 (1.0-3.8)	0.03
1310-2011	107	48 %	810	40 %	1.4 (1.1-1.9)	0.01

**Fig. 2:**
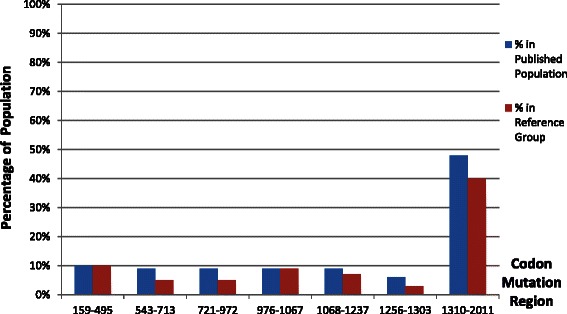
Bar graph comparing frequency of codon mutations in both the published cases of desmoid tumors and the reference population of FAP

When comparing our desmoid tumor database to previously published APC gene mutations, our database was able to be broken down into 7 regions of the APC gene. This was based on previously published data that grouped APC mutations into smaller groups by location, based in turn, on the method of testing for APC mutation [4]. The odds ratios were between 0.9-2.0 [Table 1]. Mutations in the APC gene from codon 1310 to 2011 were the most common region encompassing 48 % of published desmoids cases and 40 % of our reference population [Fig. 2]. This region additionally had a slightly elevated odds ratio of 1.4 (1.1 – 1.9) that was statistically significant (*p* = 0.01). This region includes the codon region of 1444 and 1578 which has been previously suspected to have increased risk of desmoid tumors [16]. However, when comparing published desmoid cases to the reference population, we observed that another codon region, 543-713, had an even higher odds ratio for desmoid tumors of 2.0 (1.2 – 3.3) that was also statistically significant (*p* = 0.01). None of the remaining 5 codon regions had statistically significant odds ratios. The most common point mutation found in our affected patients was codon 1309 (13.1 %), but it was also the most commonly found mutation in our reference population (12.9 %). The odds ratio was 1.0 (0.6 – 1.5) which suggested no increased risk when compared to our reference population. There was an addition codon mutation region with an odds ratio of 2.0 (1.0 – 3.8) at codons 1256-1303 that was statistically significant (*p* = 0.03). However, as the confidence interval included 1.0 it could not be reliably confirmed as being at higher risk for desmoid tumor formation.

## Discussion

We believe that this is the largest group of pooled cases that includes desmoid tumors and APC gene mutations reported to date. The mean age of onset (30.0 years) tallies with previously reported data. There was also a female predominance for desmoid tumors which has been reported in thyroid carcinoma in FAP as well [14]. However, this is the first analysis that attempts to define the actual risk of desmoid tumors based on specific APC gene mutations while adjusting for the frequency distribution of APC mutations in an FAP reference population, in an attempt to obviate bias toward more common mutations. Our group had previously performed a similar study regarding thyroid cancer and FAP [14]. The observations from that study also revealed the need to re-examine other recommendations on phenotype-genotype correlations that are of even greater clinical relevance such as desmoid tumors.

Previous authors have noted that desmoids were most strongly associated with APC gene mutations from codons 1309-1580. Our data does indicate that mutations in codons 1310-2011 are associated with an increased risk of desmoid tumors; however, codon 1309 itself does not seem to confer an increased risk of desmoid tumor alone. Individuals harboring mutation at codon 1309 may be reported as associated with more extra-intestinal manifestations by virtue of being one of the more prevalent mutations, but does not appear to be associated with an increased risk of desmoid tumor formation when using our methods. We made a similar observation in our earlier work exploring the genotypic associations between FAP and thyroid cancer [14]. This argues that higher risk codons lay further down the 3′ end of this coding region. Lastly, by comparing published data to a reference population, we identified an even higher risk region within 543-713 that was not previously recognized for desmoid tumors.

Genotype-directed disease surveillance has repeatedly been proposed as a potential strategy geared toward the detection of early intestinal and extra-colonic malignancy in FAP. Whether the specific APC mutation prompts different desmoid tumor surveillance techniques and schedules in certain groups of patients has not been previously determined. A recent review of extra-abdominal desmoid tumors did not reveal any specific surveillance guidelines much less one based on genotype [15]. These results could be used in future research to better understand the pathophysiology of desmoid tumor formation or to develop surveillance protocols for targeted codon mutations of the APC gene.

This study has several limitations; it is a pooled meta-analysis of a heterogeneous group of publications ranging from registry based reports, case series, and case reports. Although efforts were made to exclude duplicate studies through careful scrutiny of publications from the same author, group, or center we cannot be sure that individual patients were not included in more than one registry or seen at more than one center resulting in over-representation in the final dataset. This appears to have been unlikely given that duplicate identical clinical data could not be found in the final dataset. The study also necessarily suffers from the inclusion of studies over a period of time during which testing modalities for APC mutations has changed. For example, some studies based their results on protein truncation tests which provided a range of mutations, but did not reveal specific point mutations. Other studies based their results on sequencing which revealed specific codon mutations. This resulted in an accrual of data that can be challenging to compare. It is noteworthy that our search revealed only 274 published patients with FAP, a specified APC gene mutation, and desmoid tumors. This underscores the need for a common resource for the pooling of mutation analysis and clinical features of this and other relatively rare polyposis syndromes through expanded use of registries.

## Conclusion

Our study reiterates the importance of continued clinical vigilance for desmoid tumor disease. Building upon earlier studies; however, we emphasize the increased risk in individuals with mutations at both the 5′ (543-713) and 3′ (1310-2011) regions of the APC gene. It is suggested that these patients might benefit from future research to develop targeted surveillance programs in an effort to reduce morbidity and mortality from desmoids tumors in the setting of FAP.

## Additional file

Additional file 1:
**Prisma 2009 Checklist.**
